# Elevated serum creatinine levels and risk of cognitive impairment in older adults with diabetes: a NHANES study from 2011-2014

**DOI:** 10.3389/fendo.2023.1149084

**Published:** 2023-10-12

**Authors:** Yanhua Xiao, Veda Devakumar, Liyan Xu, Lei Liu, Hanyou Mo, Xuezhi Hong

**Affiliations:** ^1^Department of Rheumatology and Immunology, Affiliated Hospital of Guilin Medical University, Guilin, China; ^2^Hiller Research Unit, University Hospital Düsseldorf, Medical Faculty of Heinrich Heine University, Düsseldorf, Germany; ^3^Department of Orthopaedics, Tianjin Hospital, Tianjin, China; ^4^Department of Rheumatology and Immunology, The First Affiliated Hospital of Guangxi Medical University, Nanning, China

**Keywords:** serum creatinine, kidney injury, cognitive impairment, diabetes, NHANES

## Abstract

**Background:**

The brain and kidney have similar microvascular structure, which makes them susceptible to certain common pathophysiological processes. In this study, we examined several indicators of kidney injury/function associated with cognitive function in older diabetic patients in the hope of finding effective markers for detecting cognitive impairment (CI).

**Methods:**

A total of 2209 older participants (aged ≥60 years) from the 2011-2014 National Health and Nutrition Examination Survey (NHANES) were analyzed for the association between diabetes and CI using a multiple linear regression analysis model. Using the same approach, we also analyzed the relationship between indicators of kidney injury/function and cognitive function (Animal Fluency Test, Digit Symbol Substitution Test) in the diabetic population.

**Results:**

Diabetes was associated with CI. In age-adjusted model, older diabetics performed significantly poorer on tests of cognitive function compared to normoglycaemic individuals (1.145 points lower on the Animal Fluency Test (*P* = 0.005) and 7.868 points reduced on the Digit Symbol Substitution Test (*P* < 0.001)). In diabetics, we found elevated serum creatinine (SCr) (especially at SCr≥300uM) was associated with lower scores on cognitive function tests after strict adjustment for potential influences on cognitive function. While, albumin/creatinine ratio (ACR) was only associated with Digit Symbol Substitution score (DSS) not Animal Fluency score (AFS), and estimated glomerular filtration rate (eGFR) was only associated with CI (AFS and DSS) at the end-stage renal disease.

**Conclusion:**

SCr, as a sensitive indicator of kidney injury, was significantly associated with CI and can potentially be used as an effective marker for screening CI in older diabetics.

## Introduction

Type 2 diabetes mellitus (T2DM) is a common metabolic disease characterized by hyperglycemia, insulin resistance, and a low-grade chronic systemic inflammatory response, which may lead to a variety of secondary complications such as hypertension, cardiovascular disease, nephropathy, cerebrovascular disease and neuropathy. In recent years, cognitive impairment (CI) has also been demonstrated as a complication of T2DM and associated with cerebrovascular disease and neuropathy (1, 2). CI is characterized by problems with remembering, learning new things, concentrating or making decisions. In mild cognitive impairment (MCI), the individual remains functionally independent, while in dementia, the CI is severe enough to compromise social and/or occupational functions (3). Cross-sectional studies have shown that the risk of MCI and dementia was increased by 20-70% in T2DM compared to the normoglycemic population (4, 5). The cross-sectional association was further supported by several other studies. For example, Mark et al. found T2DM is associated with a 1.5–2.5-fold increased risk of dementia (6). A recent study also displayed that the prevalence of CI was 30% in those with normoglycaemia and 47% in those with hypoglycaemia (7). Meanwhile, data from a large US veterans’ registry showed that among older diabetics, the combined prevalence of dementia and MCI was 31.04%, whereas among normoglycaemia it was only 16.88% (7). Although, numerous studies have revealed CI in diabetics especially in older subjects (8); however, the exact mechanism of this condition remains unclear. Therefore, investigating reliable biomarkers and therapeutic targets for CI associated with diabetes is crucial for the prompt treatment and improved prognosis of diabetic patients.

In the last decade, the interaction between kidney and brain has generated a great interest, leading to numerous epidemiological and pathophysiology investigations. Theoretically, the brain and kidney have a similar microvascular structure and are vulnerable to hemodynamic fluctuations (3, 9). Both renal vascular and cerebrovascular beds are vulnerable to atherosclerotic risk factors such as aging, hypertension, hyperlipidemia and diabetes. Recent studies suggested that chronic kidney disease (CKD) is closely associated with Alzheimer’s disease (AD), stroke and cerebrovascular disease (10, 11). CI is associated with kidney function, especially in the end-stage renal disease (ESRD) (12). Among patients with ESRD, 16–38% of them suffered from memory loss, executive difficulties or speech impairment (13). Similarly, previous studies also shown that with each 10 ml/min/1.73 m^2^ decrease in estimated glomerular filtration rate (eGFR) among CKD patients, the odds of CI increase by 10-12% (11). Interestingly, a study has demonstrated that higher Serum creatinine (SCr) is associated with vascular dementia in people with good health, suggesting that dementia may occur at an early stage of kidney disease (14). And it makes us more interested in further investigating the association between markers of kidney injury and CI among diabetics.

Although several studies have linked renal disease markers to an increased risk of CI, they have been limited to individuals with kidney disease, particularly ESRD (11, 14–16). Therefore, in this study, we investigated the relationship between renal disease markers (eGFR, blood urea nitrogen [BUN], albumin/creatinine ratio [ACR] and SCr) and cognitive function in older diabetics from National Health and Nutrition Survey (NHANES). Our current study suggests a graded association between SCr and cognitive function, especially at ≥300uM. In addition, eGFR <15 mL/min/1.73 m2 and ACR ≥30ug/mg were also associated with cognitive dysfunction.

## Methods

### Subjects and study design

**Figure 1:**
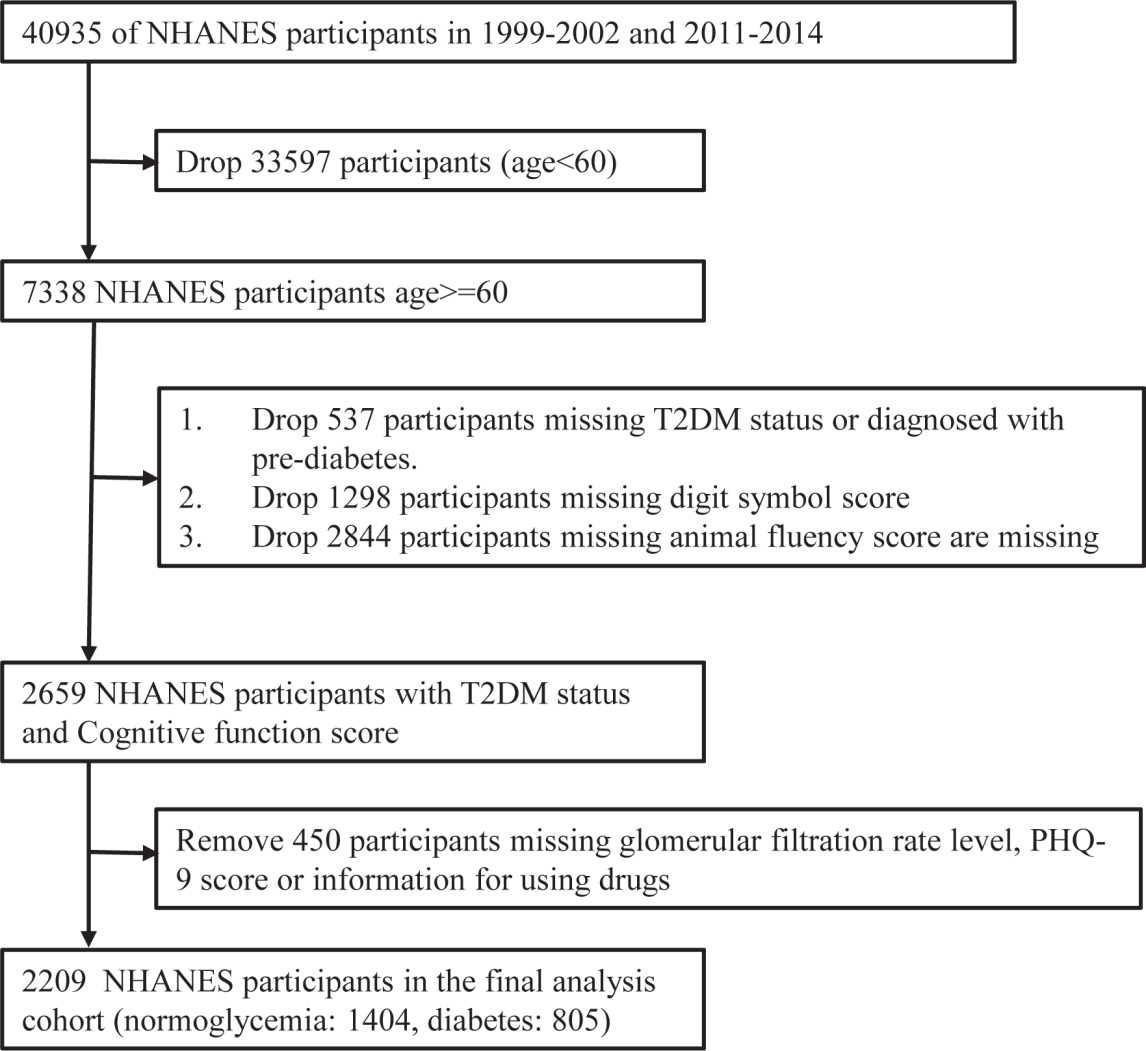
A flow chart of participant selection.

Data were collected from 2 cycles (2011-2012 and 2013-2014) of the NHANES. NHANES includes interview, examination and laboratory data collected from a complex, multi-stage, stratified, subgroup probability sample of civilian, non-institutionalized persons. A total of 19931 participants participated in the 2011-2012 and 2013-2014 demographic data surveys. From these, participants aged ≥60 years (3632 in total) were selected to examine the relationship between diabetes and cognitive function in the elderly. 334 elderly participants were diagnosed with pre-diabetes (190 participants were diagnosed with impaired glucose tolerance and 144 had impaired fasting glucose), 583 were missing a Digit Symbol Score (DSS) and 56 were missing an Animal Fluency Score (AFS). Therefore, only 2659 NHANES participants had diabetes status (Normoglycemia or DM) and cognitive function scores (DSS and AFS). We also removed data from 450 participants who were missing eGFR, patient health questionnaire-9 (PHQ-9) score or information for using drugs. Thus, there were 2209 participants in the final analysis cohort (**Figure 1**).

### T2DM

Diabetes status groups were defined as follows: (1) doctor told you have diabetes, (2) Use of diabetes medication or insulin, (3) fasting plasma glucose (mmol/l) ≥7.0, (4) random blood glucose (mmol/l) ≥ 11.1, (5) two-hour Oral Glucose Tolerance Test blood glucose (mmol/l) ≥11.1, (6) Hemoglobin A1c (HbA1c) > 6.5%. (1), (2) or (3) can be directly diagnosed as diabetes. A diagnosis of diabetes can also be confirmed by satisfying two or three of conditions in (4), (5) and (6). During the mobile examination center (MEC) visit, a phlebotomist obtained blood samples using a standardized protocol. Homeostatic model assessment of insulin resistance (HOMA-IR) is an indicator of insulin resistance and can be calculated using the following formula: fasting serum insulin (mIU/L) * fasting plasma glucose (mmol/L)/22.5.

### Cognitive function test

The Animal Fluency Test (AFT) examines categorical verbal fluency which is a component of executive functioning. Participants are asked to name as many animals as they can in one minute. One point was awarded for each animal named. In NHANES, participants were first asked to name three items of clothing, which is another verbal fluency category, as a practice test. If participants could not name three outfits, they could not continue with the animal fluency exercise. The AFS was scored on a scale of 1-40, with higher scores indicating better cognitive function.

The Digit Symbol Substitution Test (DSST) is a performance module of the Wechsler Adult Intelligence Scale (WAIS III) that relies on processing speed, sustained attention and working memory. The exercise was performed using a paper table which had a key at the top containing 9 numbers paired with symbols. Participants had 2 minutes to copy the corresponding symbols in the 133 boxes adjacent to the numbers. The score is the total number of correct matches. Before participants start the main test, a mock test is administered. In NHANES, participants will not proceed if they cannot match the symbols to the numbers correctly on their own in the pre-test exercise. Details of the scoring can be found in the data file for the 1999-2000 NHANES CFQ questionnaire (https://wwwn.cdc.gov/Nchs/Nhanes/1999-2000/CFQ.htm).

### Clinical data collection

Participants self-reported their age, gender, race/ethnicity, education, and family income and income poverty index (IPR) at the time of the interview. Meanwhile, medical history (i.e., hypertension, coronary heart disease, anemia, angina, asthma, renal failure, hyperlipidemia, stroke and smoking) and physical measurements (i.e., blood pressure, weight and height) were collected from the participants by trained technicians. Among these, body mass index (BMI) was defined as a person’s weight divided by the square of his/her height [weight (kg)/height (m^2^)].

### Laboratory measurements

Collect participants’ fast plasma glucose, fast plasma insulin, HbA1c, neutrophils, lymphocytes, platelets, monocytes, SCr, BUN, uric acid (UA), urine albumin, triglycerides (TG), high density lipoprotein (HDL), low density lipoprotein cholesterol (LDL), alkaline phosphatase (ALP), aspartate aminotransferase (AST), gamma glutamyl transferase (GGT), lactate dehydrogenase (LDH), albumin, globulin. Atherogenic index of plasma (AIP) [Log (Triglycerides/HDL-Cholesterol)] is an index of atherosclerosis in plasma. Based on the relevant research, systemic immune inflammation index (SII), system inflammation response index (SIRI), neutrophil-to-lymphocyte ratio (NLR), Platelet-to-lymphocyte ratio (PLR) and monocyte-to-lymphocyte ratio (MLR) were then calculated. SII= neutrophil/lymphocyte*Platelet, SIRI= neutrophil *Monocyte/lymphocyte, NLR= neutrophil/lymphocyte, PLR=Platelet/lymphocyte, MLR=Monocyte/lymphocyte.

### Statistical analysis

All analyses were conducted using weighted samples and considered stratification and clustering designs to produce estimates applicable to the US population. All analyses were conducted in R, version 4.2.1. Descriptive statistics were used to describe the characteristics of the subjects (weighted means and 95% confidence interval values were used for continuous variables, while quantities and percentages were used for categorical variables). Clinical biochemical parameters were compared between the normoglycaemic and diabetic populations using a t-test. Differences in cognitive function scores between normoglycaemic and diabetic populations were compared using a multiple linear regression analysis with or without adjustment for relevant parameters. To assess the association between eGFR, BUN, ACR, SCr and cognitive function in older adults with diabetes, we used multivariate analysis adjusting for age, gender, ethnicity, education, BMI, depression, drug use, and some underlying diseases that might affect vasculature. Linear regression was used for continuous outcomes. Statistical tests were considered significant at *P* < 0.05.

## Results

### Demographic, clinical and cognitive characteristics between normoglycemia and diabetes population

The 2209 NHANES participants represent 41.4 million noninstitutionalized residents of the United States. Among 2209 participants, 1404(63.6%) were normoglycemia and 805(36.4%) were DM (**Supplementary Table 1**). There was a greater percentage of black and Mexican American, and a lower proportion of women, in those with diabetes compared to those with normoglycemia. Interestingly, highly educated participants had a lower incidence of diabetes than those with less education. Similar to these results, the prevalence of diabetes decreased among the population with increasing IPR (**Supplementary Table 1**). In addition, there was a higher prevalence of hypertension, CKD, congestive heart failure, hyperlipidemia, depression and stroke among those with diabetes compared to those with normoglycemia.

To further understand the effects of diabetes on health, laboratory test results such as inflammatory indicators, lipid markers, liver function parameters, kidney function indicators, and cognitive function test scores were collected. In **Table 1**, we found biomarkers (SII, NLR, SIRI) used to assess overall inflammatory status were significantly upregulated in the diabetic population. Meanwhile, persons with diabetes also had poorer lipid profiles; for example, HDL was considerably lower in diabetics, while the AIP, BMI and TG were significantly higher than those in normoglycemic individuals. Interestingly, diabetes has no significant effect on liver function, but GGT and globulin were slightly elevated in the diabetic population. In addition, markers of kidney injury/function (such as BUN, eGFR, ACR and SCr) and UA were significantly higher among diabetics compared to the normoglycaemic population. From **Table 1**, we also observed that AFS (diabetics 17.3 vs. normoglycaemic 18.6, *P*=0.002) and DSS (diabetics 46.5 vs. normoglycaemic 55.2, *P*<0.001) were significantly decreased in the diabetic population. That means cognitive function among diabetics may be impaired compared to normoglycaemic subjects.

**Table 1:** Cognitive function scores and clinical characteristics of diabetic patients.

	Normoglycemia	DM	*P*
Fast glucose (mmol/L)	5.4(5.4,5.4)	7.9(7.5,8.2)	<0.001
Refrige glucose (mmol/L)	5.3(5.2,5.4)	7.8(7.4,8.2)	<0.001
Fast insulin (pmol/L)	55.1(50.9, 59.3)	109.9(99.5,120.2)	<0.001
HbA1c (%)	5.6(5.5,5.6)	6.9(6.8,7.1)	<0.001
HOMA-IR	2.2(2.1,2.4)	7.0(5.9,8.0)	<0.001
BMI	27.8(27.4,28.2)	31.9(31.0,32.7)	<0.001
SII	551.8(525.5,578.0)	613.1(578.1,648.1)	0.006
NLR	2.4(2.3,2.5)	2.8(2.7,3.0)	<0.001
PLR	132.6(128.1,137.1)	129.6(124.4,134.9)	0.287
SIRI	1.4(1.3,1.4)	1.6(1.5,1.7)	<0.001
MLR	0.3(0.3,0.3)	0.3(0.3,0.3)	0.376
SCr (umol/L)	83.5(82.3, 84.7)	96.3(89.5,103.2)	0.001
BUN (mmol/L)	5.6(5.5,5.7)	6.3(6.0,6.5)	<0.001
eGFR (mL/min/1.73m^2^)	74.7(73.8,75.6)	70.6(68.8,72.4)	<0.001
UA (umol/L)	326.2(321.7,330.6)	351.3(341.6,360.9)	<0.001
HCO3- (mmol/L)	25.8(25.6,26.0)	25.3(25.0,25.6)	0.002
ALT (U/L)	21.6(20.7,22.5)	23.6(22.0,25.1)	0.044
AST (U/L)	24.8(24.2,25.4)	25.4(24.1,26.7)	0.471
TG (mmol/L)	1.7(1.6,1.7)	2.1(1.9,2.2)	<0.001
Cholesterol (mmol/L)	5.1(5.0,5.2)	4.5(4.4,4.6)	<0.001
HDL (mmol/L)	1.5(1.5,1.6)	1.2(1.2,1.3)	<0.001
LDL (mmol/L)	3.0(2.9,3.1)	2.6(2.5,2.7)	<0.001
Albumin (g/dl)	4.2(4.2,4.3)	4.2(4.1,4.2)	<0.001
Globulin (g/dl)	2.7(2.7,2.7)	2.8(2.7,2.8)	<0.001
GGT (IU/L)	23.0(21.7,24.3)	28.9(26.0,31.8)	<0.001
AIP	-0.2(-0.2, -0.1)	0.1(0.0, 0.1)	<0.001
ALP (IU/L)	67.0(65.2,68.7)	69.2(66.7,71.7)	0.19
LDH (U/L)	132.5(130.1,135.0)	129.9(127.3,132.5)	0.102
Animal fluency score	18.6(18.1,19.1)	17.3(16.7,17.8)	0.002
Digit symbol score	55.2(53.8,56.6)	46.5(44.6,48.4)	<0.001

HbA1c, glycated hemoglobin A1c; HOMA-IR, homeostatic model assessment of insulin resistance; BMI, body mass index; SII, systemic immune inflammation index; NLR, neutrophil-to-lymphocyte ratio; PLR, Platelet-to-lymphocyte ratio; SIRI, system inflammation response index; MLR, monocyte-to-lymphocyte ratio; SCr, serum creatinine; BUN, blood urea nitrogen; eGFR, estimated glomerular filtration rate; UA, uric acid; ALT, alanine aminotransferase; AST, aspartate aminotransferase; TG, triglycerides; HDL, high-density lipoprotein; LDL, low-density lipoprotein; GGT, gamma glutamyl transferase; AIP, atherogenic index of plasma [Log (Triglycerides/HDL-Cholesterol)] is an index of atherosclerosis in plasma; AIP, atherogenic index of plasma [log10(TG/HDL-C)], a higher AIP value indicates a higher risk of atherosclerosis; LDH, lactate dehydrogenase.

### Correlation of cognitive function scores with diabetes

To further analyze the correlation between cognitive function and diabetes status, we used univariate linear regression and multivariable linear regression model. On univariate linear regression analysis, we found a 1.347 reduction in AFS and an 8.643 reduction in DSS in diabetics compared to the normoglycaemic population (**Table 2**). Since age is an important factor affecting cognitive function, we further analyzed the relationship between cognitive function and diabetes after adjusting for age. After adjusting by age, the AFS of diabetics decreased by 1.145 and DSS decreased by 7.868 compared to normoglycaemic subjects. In addition, we considered that gender, ethnicity, education, IPR, hypertension, chronic kidney disease (CKD), heart failure, hyperlipidaemia, smoking, depression, drug use and stroke may also affect cognitive function scores. Therefore, after adjusting for independent variables by multicollinearity analysis, we generated 4 models. Model 1 where we only adjusted for demographic parameters (ethnicity, age, sex, education), while model 2 was based on model 1 and further adjusted for hypertension, CKD, congestive heart failure, hyperlipidemia, stroke, smoke, BMI. Considering the multicollinearity problem, model 3 was added with depression and drug use in model 1, while model 4 was added with stroke, hypertension, hyperlipidaemia and CKD in model 3. In model 1, the AFS of the diabetic population decreased by 0.303 compared to the normoglycaemic population, while the DSS decreased by 4.259. Meanwhile in Model 2, the diabetic population showed a 3.710-point decrease in DSS compared to the normoglycaemic population (Tabel 2). Here, we also found a 3.609-point decrease in DSS among the diabetic population compared to the normoglycaemic population in model 3 and a 3.502-point decrease in model 4. According to these results, it is further clarified that the diabetic population shows reduced cognitive function compared to the normoglycaemic population. In the results of Model 4, besides diabetes we also found age, ethnicity, education, smoke, depression, hyperlipidemia as well as CKD were independent risk for the development of CI (**Supplementary Table 2**).

**Table 2:** Multivariate analysis of cognitive function scores and diabetes status in NHANES 2011-2014.

Models	animal fluency score			digit symbol score		
	Estimate	SE	*P*	Estimate	SE	*P*
Unadjusted	-1.347	0.397	0.002	-8.643	1.298	<0.001
Age adjusted	-1.145	0.377	0.005	-7.868	1.152	<0.001
Model 1	-0.303	0.357	0.404	-4.259	0.880	<0.001
Model 2	0.062	0.306	0.843	-3.710	1.013	0.002
Model 3	-0.082	0.354	0.819	-3.609	0.939	0.001
Model 4	0.130	0.317	0.688	-3.502	1.053	0.005

Values are expressed as Estimate and Std Error unless otherwise indicated.

Model 1 was adjusted by age, sex, race/ethnicity and education;

Model 2 was adjusted by model 1, hypertension, CKD, congestive heart failure, hyperlipidemia, stroke, smoke, BMI;

Model 3 was adjusted by model 1, depression, drug use, BMI, smoke;

Model 4 was adjusted by model 1, depression, drug use, BMI, smoke, stroke, hypertension, hyperlipidemia, and CKD.

Normoglycaemia group set as reference group.

### Elevated serum creatinine levels associated with impaired cognitive function in diabetic populations

We have found that diabetic populations have impaired cognitive function compared to normoglycemic populations. Next, we would like to further investigate the clinical indicators associated with cognitive impairment in diabetic individuals. Firstly, we analyzed demographic characteristics and found that age, education, ethnicity and IPR all had an impact on AFS and DSS in the diabetes population. However, gender only had a significant effect on AFS, but not on DSS (**Supplementary Table 3**). We then further analyzed the impact of diabetic complications on cognitive function. CKD, depression and anemia can lead to decreased AFS and DSS in diabetic populations, whereas stroke and heart failure lead to only lower DSS in people with diabetes (**Supplementary Table 3**). Additionally, we analyzed clinical biochemical indicators and BMI. In **Table 3**, we observed a significant inverse relationship between renal injury/function indicators (eGFR, BUN, SCr) and cognitive function scores in the crude model (*P*<0.001). That was, cognitive function scores decreased with increasing levels of indicators of renal dysfunction. The results also showed that in the diabetic population, ACR≥300mg/g, eGFR<15mL/min/1.73m^2^, and SCr≥200uM were associated with worse performance on both cognitive tests (DSS and AFS). Furthermore, ACR (30mg/g-300mg/g) and eGFR(15-30mL/min/1.73m^2^) were found to be only correlated with DSS performance (**Table 3**).

**Table 3:** The relationship between clinical indicators and cognitive function scores in diabetic populations.

unadjusted	animal fluency score			digit symbol score		
	Estimate	SE	*P*	Estimate	SE	*P*
NLR	-0.054	0.105	0.613	-0.123	0.221	0.584
PLR	-0.001	0.004	0.889	0.009	0.011	0.424
SII	<0.001	0.001	0.414	0.001	0.001	0.476
SIRI	0.054	0.175	0.759	0.074	0.468	0.876
MLR	-2.009	1.511	0.193	-2.432	4.570	0.598
eGFR (mL/min/1.73m2)	0.045	0.012	0.001	0.155	0.036	<0.001
SCr (umol/L)	-0.011	0.003	0.001	-0.052	0.010	<0.001
BUN (mmol/L)	-0.275	0.075	0.001	-0.976	0.216	<0.001
UA (umol/L)	-0.002	0.003	0.542	0.001	0.010	0.879
Fast glucose (mmol/L)	-0.152	0.127	0.240	-0.174	0.556	0.756
Refrige glucose (mmol/L)	-0.143	0.095	0.145	-0.348	0.267	0.202
Fast insulin (mmol/L)	0.002	0.002	0.378	0.008	0.010	0.420
HbA1c (%)	-0.377	0.233	0.116	-0.854	0.749	0.263
ALT (U/L)	0.003	0.023	0.907	0.043	0.058	0.463
AST (U/L)	-0.014	0.018	0.419	-0.039	0.032	0.225
ALP (IU/L)	-0.010	0.013	0.433	-0.072	0.038	0.066
Albumin (g/dl)	2.843	0.655	<0.001	8.122	1.919	<0.001
Globulin (g/dl)	-2.029	0.526	0.001	-6.094	1.684	0.001
GGT (IU/L)	0.002	0.008	0.772	-0.005	0.024	0.825
Bicarbonate (mmol/L)	0.072	0.121	0.557	-0.353	0.205	0.095
TG (mmol/L)	0.115	0.275	0.678	0.474	0.881	0.594
Cholesterol (mmol/L)	0.178	0.184	0.343	-0.090	0.652	0.891
HDL (mmol/L)	-1.021	0.699	0.154	-1.495	2.418	0.541
BMI	0.053	0.041	0.207	0.341	0.126	0.011
AIP	1.679	1.250	0.189	4.328	4.440	0.337
HOMA_IR	0.017	0.029	0.568	0.077	0.139	0.584
LDL (mmol/L)	0.117	0.335	0.730	-0.568	1.014	0.580
LDH (IU/L)	-0.036	0.009	<0.001	-0.089	0.030	0.006
ACR<30 mg/g	Reference			Reference		
ACR 30-300mg/g	-1.310	0.657	0.055	-4.926	1.641	0.005
ACR ≥300mg/g	-3.925	0.951	<0.001	-14.899	1.812	<0.001
eGFR ≥90mL/min/1.73m^2^	Reference			Reference		
eGFR 60-90mL/min/1.73m^2^	0.050	0.615	0.935	-1.742	2.146	0.424
eGFR 30-60mL/min/1.73m^2^	-1.314	0.811	0.117	-5.143	2.566	0.055
eGFR 15-30mL/min/1.73m^2^	-3.692	1.856	0.057	-12.698	4.321	0.007
eGFR <15mL/min/1.73m^2^	-4.480	0.640	<0.001	-22.879	2.672	<0.001
SCr <100uM	Reference			Reference		
SCr 100-200 uM	-0.850	0.670	0.214	-3.028	2.006	0.142
SCr 200-300 uM	-4.965	1.001	<0.001	-14.302	3.008	<0.001
SCr ≥300uM	-3.551	0.834	<0.001	-20.500	2.242	<0.001

Values are expressed as Estimate and Std Error (SE) unless otherwise indicated. ACR, albumin/creatinine ratio.

Then we used multiple linear regression models to identify independent factors associated with impaired cognitive function in diabetic population. According to the results of the univariate analysis, both cognitive function scores were only statistically significant with SCr, the marker of kidney injury, after adjusting for potential confounders (**Table 4**). Specifically, in model 2, after adjusting for age, gender, education, ethnicity, BMI, congestive heart failure and stroke, we were surprised to find that only SCr values had a significant effect on AFS. We then stratified eGFR, ACR and SCr and found that ACR ≥300mg/g, eGFR <15 mL/min/1.73 m2, and SCr ≥300uM were all associated with decreased DSS and AFS scores. While in model 3, where we adjusted for age, gender, education, ethnicity, depression, drug use, stroke, hypertension, hyperlipidemia, congestive heart failure and BMI, we found that for each 1 mM increase in SCr, the DSS decreased by 0.028 points (*P*=0.003), AFS decreased by 0.006 points (*P*=0.002). Meanwhile, we also found a 16.885-point decrease in DSS and a 2.503-point decrease in AFS for those with SCr ≥300uM compared to those with SCr<100uM, after adjustment for age, sex, education, and other potential confounders in model 3. Previous studies have shown that non-steroidal anti-inflammatory drugs (NSAIDs) can affect renal and cognitive function, so in model 4 we replaced drug use with NSAIDs use status (no, yes, other), while the other independent variables remained the same as in model 3. The results of model 4 showed minimal changes in the coefficients of the individual variables compared to model 3 (**Table 4**). As diuretics can also affect renal and cognitive function, we performed a sensitivity analysis with the same comparison on a sample excluding individuals with diuretics administration (**Supplementary Table 4**). Similar results were obtained in model 3 when subjects using diuretics or with missing drug names were excluded.

**Table 4:** Relationship between serum creatinine, blood urea nitrogen, estimated glomerular filtration rate, albumin/creatinine ratio and cognitive function scores in diabetic populations.

Model	animal fluency score			digit symbol score		
	Estimate	SE	*P*	Estimate	SE	*P*
Model 1						
BUN	-0.159	0.079	0.055	-0.400	0.183	0.039
eGFR	0.023	0.013	0.102	0.052	0.036	0.159
SCr	-0.009	0.003	0.004	-0.035	0.008	<0.001
eGFR ≥90mL/min/1.73m^2^	reference			reference		
eGFR 60-90mL/min/1.73m^2^	0.561	0.694	0.431	1.614	1.473	0.289
eGFR 30-60mL/min/1.73m^2^	-0.237	0.827	0.779	0.761	1.783	0.675
eGFR 15-30mL/min/1.73m^2^	-0.748	1.613	0.649	-3.476	3.513	0.337
eGFR <15 mL/min/1.73m^2^	-3.129	1.159	0.016	-17.285	5.073	0.004
ACR<30 mg/g	reference			reference		
ACR 30-300 mg/g	-0.703	0.507	0.183	-2.241	1.556	0.167
ACR≥300 mg/g	-2.126	0.955	0.039	-5.935	2.136	0.012
SCr <100uM	reference			reference		
SCr100-200uM	-0.805	0.540	0.151	-0.583	1.405	0.682
SCr200-300uM	-2.094	1.110	0.072	-2.691	1.884	0.167
SCr≥300uM	-3.548	1.087	0.004	-20.033	4.028	<0.001
Model 2						
BUN	-0.115	0.073	0.132	-0.282	0.192	0.157
eGFR	0.020	0.013	0.149	0.038	0.038	0.331
SCr	-0.008	0.002	0.004	-0.032	0.008	0.001
eGFR≥90mL/min/1.73m^2^	reference			reference		
eGFR60-90mL/min/1.73m^2^	0.551	0.665	0.418	1.810	1.540	0.255
eGFR30-60mL/min/1.73m^2^	-0.249	0.797	0.758	1.017	1.806	0.580
eGFR15-30mL/min/1.73m^2^	-0.762	1.640	0.648	-3.024	3.461	0.394
eGFR<15 mL/min/1.73m^2^	-3.160	1.107	0.011	-16.046	4.532	0.002
ACR<30 mg/g	reference			reference		
ACR 30-300 mg/g	-0.659	0.510	0.211	-2.608	1.543	0.106
ACR≥300 mg/g	-2.091	0.959	0.041	-6.229	2.031	0.006
SCr <100uM	reference			reference		
SCr100-200uM	-0.790	0.548	0.166	-0.086	1.446	0.953
SCr200-300uM	-1.410	1.068	0.203	-2.326	2.189	0.301
SCr≥300uM	-3.662	1.021	0.002	-19.364	4.128	<0.001
Model 3						
BUN	-0.095	0.067	0.174	-0.240	0.194	0.235
eGFR	0.015	0.012	0.237	0.029	0.035	0.420
SCr	-0.006	0.002	0.026	-0.028	0.008	0.003
eGFR≥90mL/min/1.73m^2^	reference			reference		
eGFR60-90mL/min/1.73m^2^	0.454	0.600	0.464	1.676	1.441	0.268
eGFR30-60mL/min/1.73m^2^	-0.216	0.788	0.789	1.046	1.770	0.565
eGFR15-30mL/min/1.73m^2^	-0.501	1.302	0.707	-2.135	2.645	0.435
eGFR<15mL/min/1.73m^2^	-2.470	1.050	0.037	-14.423	4.072	0.004
ACR<30 mg/g	reference			reference		
ACR 30-300 mg/g	-0.321	0.454	0.492	-2.059	1.281	0.130
ACR≥300 mg/g	-1.656	0.856	0.074	-5.508	1.706	0.006
SCr <100uM	reference			reference		
SCr100-200uM	-0.541	0.491	0.290	0.371	1.455	0.803
SCr200-300uM	-1.230	0.916	0.202	-1.901	2.285	0.421
SCr≥300uM	-2.503	1.068	0.036	-16.885	4.157	0.001
Model 4						
BUN	-0.106	0.065	0.127	-0.213	0.191	0.283
eGFR	0.017	0.012	0.175	0.024	0.035	0.500
SCr	-0.006	0.002	0.019	-0.027	0.008	0.005
eGFR≥90mL/min/1.73m^2^	reference			reference		
eGFR60-90mL/min/1.73m^2^	0.456	0.613	0.473	2.002	1.410	0.183
eGFR30-60mL/min/1.73m^2^	-0.333	0.793	0.682	1.422	1.690	0.418
eGFR15-30mL/min/1.73m^2^	-0.543	1.267	0.676	-1.603	2.885	0.590
eGFR<15mL/min/1.73m^2^	-2.522	1.046	0.035	-13.968	3.891	0.004
ACR<30 mg/g	reference			reference		
ACR 30-300 mg/g	-0.289	0.437	0.520	-1.873	1.273	0.165
ACR≥300 mg/g	-1.650	0.857	0.076	-5.558	1.700	0.006
SCr <100uM	reference			reference		
SCr100-200uM	-0.655	0.448	0.170	0.658	1.430	0.653
SCr200-300uM	-1.309	0.957	0.196	-2.069	2.406	0.407
SCr≥300uM	-2.552	1.054	0.032	-16.574	3.980	0.001

Values are expressed as Estimate and Std Error unless otherwise indicated.

Model 1 was adjusted by age, sex, race/ethnicity and education;

Model 2 was adjusted by model 1, BMI, congestive heart failure and stroke;

Model 3 was adjusted by model 2, depression, drug use, hypertension and hyperlipidemia;

Model 4 was adjusted by model 2, depression, non-steroidal anti-inflammatory drugs (NSAIDs), hypertension and hyperlipidemia.

As shown in **Table 4**: (1) elevated SCr (especially at SCr≥300uM) was associated with lower scores on cognitive function tests (AFS and DSS) after strict adjustment for potential influences on cognitive function. (2) Meanwhile, after adjusting for potential confounders, an ACR≥300mg/g may also result in lower cognitive function scores, but there was no significant difference in cognitive function AFS scores in model 3 and model 4. (3) In addition, there was a significant decline only in end-stage CKD (eGFR<15 ml/min/1.73 m^2^).(4)Elevated BUN may also lead to lower cognitive function scores after adjusting for potential confounders, however, there were no significant differences in cognitive function scores in model 2, model 3 and model 4. We therefore consider, SCr might serve as a risk factor for impaired cognitive function in the diabetic population.

## Discussion

We found that compared to participants with normoglycaemia, diabetics were more likely to have CI in our study. These results were found in the DSS after adjusting for demographic characteristics and were marginally significant after adjusting for health behaviors and comorbid conditions, including hypertension, hyperlipidaemia, stroke, depression, BMI, CKD, and smoking. Interestingly, we also observed a decline in cognitive function accompanied by an increase in renal biomarkers among individuals with diabetes. However, among the several indicators of renal injury/function we examined, except eGFR (eGFR<15), only SCr remained significantly associated with cognitive function (both AFS and DSS) after adjusting for potential confounders. Our results suggest SCr could be a risk factor for CI in older diabetic subjects.

In this current study, we first examined the correlation between SCr levels and cognitive performance in diabetics and observed that SCr levels in individuals with diabetes were negatively associated with cognitive function scores (Each 1 mg/dL rise in SCr was associated with a 0.028 decrease in the DSS and 0.006 decrease in the AFS). Especially with SCr≥300uM, cognitive function scores were significantly decreased in the older diabetic population (16.885 points decrease in DSS and 2.503 points decrease in AFS compared to those with SCr<100uM). This observation is consistent with previous researches- Seliger suggested that elevated SCr in healthy population were associated with vascular dementia but not Alzheimer-type dementia, where it was observed that with each 1.0 mg/dl decrease in SCr there was a 26% increased risk of vascular dementia (95% CI = 1.02 to 1.60) (14). Kurella and Khatri also observed an increase in SCr was associated with an accelerated rate of CI (17, 18). Altogether, these indicated that SCr is strongly associated with cognitive function.

To our knowledge, SCr is a waste product formed in skeletal muscle during the metabolism of creatine phosphate and excreted by the kidney, thus the concentration is relatively stable *in vivo*. An elevated SCr concentration (for men>114.9 µmol/L, for women>97.2µmol/L) may indicate kidney injury (19). Notably, in addition to SCr being associated with cognitive function in our study, we also found other indicators of renal injury/function such as BUN, eGFR and ACR were related to cognitive function under unadjusted demographic parameters. These results may indicate a correlation between renal injury and CI in diabetics. Indeed, series of studies have shown that diabetes is associated with an increased risk of CI and kidney injury, and kidney injury can also lead to the occurrence of CI and accelerate disease progression (20–22). Moreover, several biological mechanisms could be responsible for provoking CI and exacerbating renal injury in diabetics. Specifically, the brain and kidneys, both organs with low impedance vascular beds, can react to blood pressure and flow fluctuations in a similar and unique way (23). Therefore, hypertension, cardiovascular disease and diabetes each affect the haemodynamics of the kidney and brain and contribute to kidney injury and CI (22). In addition, peripheral inflammatory markers such as SII, SIRI, NLR, and soluble adhesion molecules, were increased in diabetic patients, which can further accelerate renal and cerebrovascular damage (1, 24).Furthermore, long-term use of some drugs (e.g. NSAIDs, opiate analgesics, etc.) can also lead to kidney damage and CI (25). It has been seen that kidney injury, by itself, could also lead to an increased risk of CI. For example, amino acid metabolism disturbances (hyperhomocysteinemia), anemia, and Acid-base imbalance (HCO3^-^<22mmol/L) in CKD individuals, all of these risk factors can destroy the cerebrovascular and further influenced the function of cognitive (3, 26, 27).

Indeed, our findings support several of above mechanisms: (1) Compared to normoglycemic people, diabetics are more likely to have co-occurring dyslipidemia, heart disease, hypertension, and significantly higher levels of inflammation, which could cause kidney-cerebrovascular injury. (2) Decreased cognitive function scores were associated with the degree of anemia, showing a mild decreased AFS in the mildly anemic population compared to the non-anemic population, while the severely anemic population showed a significant decrease in both AFS and DSS. (3) Impairment of renal function was directly correlated with CI. Results indicated SCr (especially for scr≥300uM) as an independent risk factor not only for lower DSS, but also for lower AFS. However, the association between BUN and AFS in this study was not as significant as the association between BUN and DSS after adjusting for demographic data, relevant diseases and other important factors. Furthermore, eGFR was only associated with CI (both AFS and DSS) at the ESRD (eGFR<15 ml/min/1.73 m^2^). We know that eGFR is converted from SCr levels by the chronic kidney disease epidemiology collaboration, modification of diet in renal disease, or Cockcroft-Gault methods, whereas SCr can be obtained directly from laboratory results and is, therefore, more convenient and simpler than eGFR. Considering the negative correlation between SCr and CI, we suggest elevated SCr (especially for SCr ≥300uM) is a risk factor for CI.

This study has some limitations. Firstly, this was a cross-sectional study, these hypotheses cannot be proven by this study and require longitudinal data to demonstrate. Diabetics with comorbid severe CI may not be able to complete cognitive function tests, so we were lacking data on this population. In addition, the relationship between cognitive function scores and indicators of renal function was biased because patients with renal insufficiency were more likely to experience early death. Furthermore, several drugs are clinically associated with impairments in kidney and cognitive function, but the types and numbers of drugs used by patients were not detailed in this study. Other biases due to unmeasured or residual confounders cannot be excluded. Finally, this study measured cognitive function through AFS and DSS, but we were unable to accurately define the cognitive function status, such as normal, MCI and dementia, based on their scores. The relationship between medications, cognitive function and kidney function can be explored in depth in future studies.

Despite the limitations of this study, there are several strengths. Most importantly, since diabetes is more likely to be complicated by cognitive dysfunction, we focused on finding sensitive indicators associated with CI in the diabetic population. In addition, we explored the relationship between renal injury/function markers, lipid parameters, inflammatory, hepatorenal function parameters and cognitive impairment in a representative population of older Americans with diabetes, but only renal injury/function markers were significantly associated with CI. Furthermore, we adjusted for many known correlates of renal impairment and cognitive function (e.g., demographics, disease, and related clinical indicators, drug use) and observed that diabetics who tend to have higher SCr concentrations may be at greater risk for cognitive impairment for unmeasured other reasons. Additionally, we used GPOWER software to calculate the minimum required sample size, which was determined to be 184 patients for 12 predictors (28). Our study included 805 older patients with diabetes, which exceeds the minimum required sample size and should therefore be sufficient to obtain statistically significant results.

Taken together, renal injury/function indicators have been associated with reduced cognitive performance and executive function in the diabetic population. SCr, as a sensitive indicator of kidney injury, was significantly associated with CI and can potentially be used as an effective marker for screening CI in older diabetics. This may enable the appropriate selection of potential timing for intervention. However, the exact mechanism of this association requires further study to elucidate.

## Data availability statement

The original contributions presented in the study are included in the article/**Supplementary Material**. Further inquiries can be directed to the corresponding authors.

## Ethics statement

The studies involving human participants were reviewed and approved by National center for health statistics Ethics Review Board. The patients/participants provided their written informed consent to participate in this study.

## Author contributions

YX: Formal analysis (lead), writing - original draft (lead). VD: Data curation (equal), review and revise the grammar and syntax (equal). LX: Data curation (equal), investigation (equal). LL: Data curation (equal), investigation(equal). HM: Conceptualization (equal), funding acquisition (lead), supervision(lead). XH: Conceptualization (equal), funding acquisition (supporting), methodology (lead), Project administration (lead), software (lead), writing - review and editing (equal). All authors contributed to the article and approved the submitted version.
