# Supplementary material for: Elevated serum creatinine levels and risk of cognitive impairment in older adults with diabetes: a NHANES study from 2011-2014
Source: Front Endocrinol (Lausanne). 2023 Oct 12;14:1149084. doi: 10.3389/fendo.2023.1149084 (PMC10603184; doi:10.3389/fendo.2023.1149084)
Supplement: Supplementary file 1 [file DataSheet_1.pdf]

Supplementary Table1 Characteristics of the population participating in the Cognitive Functioning Score and Diabetes Status Assessment (age≥60, NHANES 2011-2014)

	Normoglycemia	Diabetes	X^2	*P*
Total	1404 (63.6%)	805(36.4%)		
Sex				
Male	651(46.4%)	421(52.3%)	6.960	0.008
Female	753 (53.6%)	384(47.7%)		
Education				
Grade school	265(18.9%)	252(31.3%)	56.097	<0.001
High School	323(23.0%)	204(25.3%)		
College	816(58.1%)	349(43.3%)		
Ethnicity				
Mexican American	761(54.2%)	330(41.0%)	38.862	<0.001
Non-Hispanic Black	293(20.9%)	223(27.7%)		
Non-Hispanic White	92(6.6%)	82(10.2%)		
Other	258(18.4%)	170(21.1%)		
Age				
60-69	786(56.0%)	428(53.2%)	7.4732	0.024
70-79	385(27.4%)	263(32.7%)		
80-89	233(16.6%)	114(14.2%)		
IPR				
<1	194(13.8%)	175(21.7%)	65.879	<0.001
1 to 2	385(27.4%)	269(33.4%)		
2 to 3	203(14.5%)	114(14.2%)		
3 to 4	170(12.1%)	100(12.4%)		
4 to 5	118(8.4%)	52(6.5%)		
>=5	334(23.8%)	95(11.8%)		
Smoke				
Never	722(51.4%)	373(46.3%)	6.5276	0.038
Former	501(35.7%)	330(41.0%)		
Now	181(12.9%)	102(12.7%)		
Hypertension				
No	484(34.5%)	153(19.0%)	58.892	<0.001
Yes	920(65.5%)	652(81.0%)		
Hyperlipidemia				
No	286(20.4%)	86(10.7%)	33.596	<0.001
Yes	1118(79.6%)	719(89.3%)		
CKD				
No	1005(71.6%)	430(53.4%)	73.376	<0.001
Yes	399(28.4%)	375(46.4%)		
Congestive heart failure				
No	1341(95.5%)	710(88.2%)	40.121	<0.001
Yes	63(4.5%)	95(11.8%)		
Coronary heart disease				

No	1312（93.4%）	695（86.3%）	30.298	<0.001
Yes	92（6.6%）	110（13.7%）		
Anemia				
Non-Anemia	1242(88.5%)	646(80.2%)	28.741	<0.001
Mild	135(9.6%)	126(15.7%)		
Moderate and Severe	27(1.9%)	33(4.1%)		
Stroke				
No	1318(93.9%)	726(90.2%)	9.5439	0.002
Yes	86(6.1%)	79(9.8%)		
Depression				
No depression	1120(79.8%)	556(69.1%)	37.012	<0.001
Mild depression	191(13.6%)	145(18.0%)		
Moderate depression	57(4.1%)	63(7.8%)		
Severe depression	36(2.6%)	41(5.1%)		
Take drug				
No	280(19.9%)	41(5.1%)	89.652	<0.001
Yes	1124(80.1%)	764(94.9%)		

IPR, income poverty index; CKD, chronic kidney disease; Patient Health Questionnaire-9 (PHQ-9) scores 5, 10, 15, and 20 represent the cut-off points for mild, moderate, moderate-severe, and severe depression, respectively. Mild anemia: hemoglobin level between 10 and 12 g/dL for adult women, and between 10 and 13 g/dL for adult men; Moderate anemia: hemoglobin level between 8 and 10 g/dL; Severe anemia: hemoglobin level below 8 g/dL.

Supplementary Table 2 Multivariate analysis of cognitive function scores and diabetes status in NHANES 2011-2014

	animal fluency score			digit symbol score		
	Estimate	SE	*P*	Estimate	SE	*P*
Normoglycemia	Reference			Reference		
DM	0.130	0.317	0.688	-3.501	1.052	0.005
Sex-Male	Reference			Reference		
Sex-Female	-0.179	0.383	0.647	4.601	0.704	<0.001
Age	-0.237	0.032	<0.001	-0.951	0.052	<0.001
Edu-Grade school	Reference			Reference		
Edu-High School	0.688	0.340	0.064	7.668	0.885	<0.001
Edu-College	3.455	0.261	<0.001	14.888	0.944	<0.001
Eth- Non-Hispanic White	Reference			Reference		
Eth- Non-Hispanic Black	-2.815	0.317	<0.001	-11.304	0.951	<0.001
Eth- Mexican American	-0.871	0.492	0.010	-10.120	1.165	<0.001
Eth-Other	-2.756	0.441	<0.001	-7.032	1.163855	<0.001
Depression-Non	Reference			Reference		
Depression-Mild	-0.647	0.312	0.059	-3.758	0.928	0.001
Depression-Moderate	-1.235	0.430	0.013	-2.659	1.913	0.188
Depression-Severe	-2.843	0.663	<0.001	-11.403	2.147	<0.001
Drug use-No	Reference			Reference		
Drug use-Yes	-0.506	0.443	0.274	-1.280	1.278	0.335
BMI	-0.016	0.025	0.533	0.032	0.076	0.682
Smoke-Never	Reference			Reference		
Smoke-Former	0.212	0.329	0.530	0.244	0.993	0.810
Smoke-Now	-0.62132	0.506	0.241	-3.540	1.286	0.016
Stroke-No	Reference			Reference		
Stroke-Yes	-0.282	0.606	0.649	-1.899	1.042	0.091
Hypertension-No	Reference			Reference		
Hypertension-Yes	-0.943	0.396	0.033	-0.503	0.819	0.550
Hyperlipidemia-No	Reference			Reference		
Hyperlipidemia-Yes	0.581	0.392	0.162	1.277	0.922	0.189
CKD-No	Reference			Reference		
CKD-Yes	-0.334	0.341	0.346	-1.850	0.609	0.009

Values are expressed as Estimate and Std Error unless otherwise indicated.

This model was adjusted by age, sex, race/ethnicity and education, depression, drug use, BMI, smoke, stroke, hypertension, hyperlipidemia, CKD.

Supplementary Table 3 Analysis of the relationship between demographic characteristics and cognitive function in diabetic populations (unadjusted)

	animal fluency score			digit symbol score		
unadjusted	Estimate	SE	*P*	Estimate	SE	*P*
Age (continuous variable)	-0.223	0.033	<0.001	-0.976	0.122	<0.001
Age 60-70	Reference			Reference		
Age 70-79	-1.886	0.493	0.001	-8.636	1.434	<0.001
Age 80-89	-3.506	0.606	<0.001	-13.681	1.920	<0.001
Sex-Male	Reference			Reference		
Sex-Female	-1.031	0.503	0.049	1.277	1.322	0.342
Edu-Grade school	Reference			Reference		
Edu-High School	0.766	0.671	0.263	11.205	1.583	<0.001
Edu-College	4.211	0.673	<0.001	18.707	1.816	<0.001
Eth- Non-Hispanic white	Reference			Reference		
Eth- Non-Hispanic black	-3.106	0.424	<0.001	-10.966	1.631	<0.001
Eth- Mexican american	-1.362	0.865	0.126	-12.517	2.335	<0.001
Eth-Other	-2.109	0.880	0.023	-7.153	2.845	0.018
IPR	0.917	0.210	<0.001	4.327	0.604	<0.001
Hypertension-No	Reference			Reference		
Hypertension-Yes	-0.620	0.566	0.281	0.084	1.722	0.961
Hyperlipidemia-No	Rreference			Reference		
Hyperlipidemia-Yes	1.031	0.942	0.282	2.500	2.622	0.348
Anemia-Non	Reference			Reference		
Anemia-Mild	-1.998	0.818	0.021	-9.544	2.136	<0.001
Anemia-Moderate and Severe	-3.633	0.772	<0.001	-6.995	3.678	0.067
Asthma-No	Reference			Reference		
Asthma-Yes	0.505	0.975	0.608	-1.192	2.447	0.630
COPD-No	Reference			Reference		
COPD-Yes	-0.126	0.928	0.893	-2.758	2.089	0.196
CKD-No	Reference			Reference		
CKD-Yes	-1.579	0.570	0.009	-5.799	1.706	0.002
Heart failure-No	Reference			Reference		
Heart failure-Yes	-1.162	0.832	0.173	-6.881	1.625	<0.001
Non-depression	Reference			Reference		
Mild depression	-1.896	0.658	0.007	-4.740	1.726	0.010
Moderate depression	-2.185	0.701	0.004	-2.384	3.479	0.499
Severe depression	-4.725	1.157	<0.001	-12.725	3.199	<0.001
Stroke-No	Reference			Reference		
Stroke-Yes	-0.737	1.112	0.512	-6.248	1.965	0.003
Non-smoke	Reference			Reference		
Smoke former	0.510	0.623	0.419	-1.238	1.653	0.460
Smoke now	0.610	0.905	0.506	-2.113	1.953	0.288
Drug use-no	Reference			Reference		
Drug use-yes	-0.842	1.722	0.628	-4.197	5.550	0.455

Supplementary Table 4 Sensitivity analysis by excluding individuals using diuretics

	All DM populations			Removed subjects using diuretics		
	Estimate	SE	*P*	Estimate	SE	*P*
**animal fluency score -model 3 +SCr**						
(Intercept)	32.812	3.599	<0.001	32.417	3.681	<0.001
SCr	-0.006	0.002	0.026	-0.006	0.002	0.032
Eth- Non-Hispanic Black	-2.814	0.451	<0.001	-2.765	0.473	<0.001
Eth- Mexican american	-0.870	0.632	0.189	-0.939	0.697	0.197
Eth-Other	-2.593	0.426	<0.001	-2.471	0.433	<0.001
Sex-Female	-0.142	0.483	0.773	-0.172	0.492	0.731
Age	-0.232	0.031	<0.001	-0.228	0.032	<0.001
Edu-High School	-0.112	0.641	0.864	-0.084	0.622	0.895
Edu-College	3.024	0.515	<0.001	3.088	0.509	<0.001
BMI	-0.001	0.048	0.976	0.002	0.049	0.976
Congestive heart failure-yes	0.172	0.744	0.820	0.121	0.736	0.872
Stroke-yes	1.221	0.807	0.151	1.193	0.820	0.166
Depression-Mild depression	-1.682	0.625	0.017	-1.683	0.627	0.017
Depression-Moderate depression	-2.422	0.540	<0.001	-2.747	0.628	0.001
Depression-Severe depression	-4.143	1.170	0.003	-4.126	1.168	0.003
Drug use-yes	0.609	1.463	0.683	0.600	1.462	0.687
Hypertension-yes	-0.373	0.569	0.522	-0.385	0.575	0.513
Hyperlipidemia-yes	0.797	0.833	0.354	0.800	0.851	0.362
**digit symbol score -model 3 +SCr**						
(Intercept)	99.811	8.679	<0.001	100.416	8.847	<0.001
SCr	-0.028	0.008	0.003	-0.027	0.008	0.005
Eth- Non-Hispanic Black	-10.016	1.491	<0.001	-10.048	1.468	<0.001
Eth- Mexican american	-9.136	1.671	<0.001	-9.312	1.609	<0.001
Eth-Other	-7.561	1.562	<0.001	-7.229	1.523	<0.001
Sex-Female	3.472	1.139	0.008	3.470	1.195	0.011
Age	-0.926	0.087	<0.001	-0.938	0.088	<0.001
Edu-High School	7.824	1.012	<0.001	7.888	0.948	<0.001
Edu-College	14.296	1.169	<0.001	14.229	1.154	<0.001
BMI	0.073	0.096	0.460	0.080	0.098	0.427
Congestive heart failure-yes	-3.127	1.141	0.015	-3.114	1.097	0.012
Depression-Mild depression	-3.666	1.345	0.016	-3.813	1.342	0.012
Depression-Moderate depression	-2.177	2.016	0.297	-3.127	1.745	0.093
Depression-Severe depression	-10.232	3.781	0.016	-10.411	3.790	0.015
Stroke-yes	-0.572	1.338	0.675	-0.466	1.354	0.736
Drug use-yes	2.100	4.183	0.623	2.183	4.181	0.609
Hypertension-yes	0.431	1.154	0.714	0.444	1.177	0.711
Hyperlipidemia-yes	2.626	2.620	0.332	2.546	2.666	0.355
**animal fluency score -model 3 +SCr (stratified analysis)**						
(Intercept)	32.286	3.641	<0.001	31.859	3.720	<0.001
SCr100-200uM	-0.541	0.491	0.290	-0.560	0.477	0.262

SCr200-300uM	-1.230	0.916	0.202	-1.230	0.967	0.226
SCr≥300uM	-2.503	1.068	0.036	-2.476	1.079	0.039
Eth- Non-Hispanic Black	-2.780	0.466	<0.001	-2.728	0.489	<0.001
Eth- Mexican american	-0.866	0.635	0.196	-0.941	0.702	0.203
Eth-Other	-2.607	0.420	<0.001	-2.487	0.427	<0.001
Sex-Female	-0.140	0.473	0.772	-0.177	0.485	0.721
Age	-0.230	0.032	<0.001	-0.226	0.033	<0.001
Edu-High School	-0.166	0.641	0.800	-0.141	0.625	0.825
Edu-College	3.032	0.515	<0.001	3.096	0.512	<0.001
BMI	-0.002	0.048	0.971	0.001	0.049	0.980
Congestive heart failure-yes	0.158	0.754	0.838	0.108	0.743	0.887
Stroke-yes	1.253	0.819	0.150	1.222	0.830	0.164
Depression-Mild depression	-1.710	0.614	0.016	-1.706	0.616	0.016
Depression-Moderate depression	-2.422	0.539	0.001	-2.741	0.621	0.001
Depression-Severe depression	-4.083	1.167	0.004	-4.058	1.163	0.004
Drug use-yes	0.587	1.453	0.693	0.581	1.451	0.695
Hypertension-yes	-0.308	0.559	0.591	-0.319	0.562	0.580
Hyperlipidemia-yes	0.788	0.832	0.361	0.791	0.851	0.370
**digit symbol score -model 3 +SCr (stratified analysis)**						
(Intercept)	99.940	8.560	<0.001	100.707	8.704	<0.001
SCr100-200uM	0.371	1.455	0.803	0.551	1.392	0.699
SCr200-300uM	-1.901	2.285	0.421	-1.745	2.369	0.474
SCr≥300uM	-16.885	4.157	0.001	-16.717	4.251	0.002
Eth- Non-Hispanic Black	-10.111	1.489	<0.001	-10.144	1.469	<0.001
Eth- Mexican american	-9.085	1.658	<0.001	-9.237	1.597	<0.001
Eth-Other	-7.407	1.597	<0.001	-7.055	1.555	0.001
Sex-Female	3.891	1.249	0.008	3.904	1.301	0.010
Age	-0.957	0.087	<0.001	-0.971	0.088	<0.001
Edu-High School	7.904	1.079	<0.001	7.972	1.018	<0.001
Edu-College	14.280	1.148	<0.001	14.217	1.152	<0.001
BMI	0.064	0.095	0.514	0.071	0.097	0.481
Congestive heart failure-yes	-3.364	1.202	0.015	-3.338	1.154	0.013
Depression-Mild depression	-3.677	1.375	0.019	-3.820	1.372	0.015
Depression-Moderate depression	-2.159	2.071	0.316	-3.125	1.791	0.105
Depression-Severe depression	-10.489	3.839	0.017	-10.697	3.868	0.016
Stroke-yes	-0.850	1.413	0.558	-0.745	1.448	0.615
Drug use-yes	1.821	4.120	0.666	1.890	4.118	0.654
Hypertension-yes	0.285	1.290	0.828	0.276	1.313	0.837
Hyperlipidemia-yes	2.605	2.631	0.340	2.530	2.675	0.362

Values are expressed as Estimate and Std Error unless otherwise indicated. After removed 22 subjects using diuretics or with missing drug names, the 783 remaining diabetic individuals were then analyzed by multivariate linear regression using model 3. SCr<100uM, Eth-Non-Hispanic white, sex-Male, Edu-Grade school, Congestive heart failure-no, Non-Depression, Stroke-No, Drug use-No, Hypertension-No and Hyperlipidemia-No set as reference group

Model 3 was adjusted by age, sex, race/ethnicity, education, BMI, congestive heart failure, stroke, depression, drug use, hypertension and hyperlipidemia.
